# Novel electrical transport properties of native Fe-Nb oxide layers leading to unilateral conductivity of a refractory metallic glass

**DOI:** 10.1016/j.heliyon.2019.e01424

**Published:** 2019-03-29

**Authors:** A.S. Trifonov, A.V. Lubenchenko, S.V. Ketov, S.V. Taskaev, D.V. Louzguine-Luzgin

**Affiliations:** a Quantum Technology Centre, Faculty of Physics, M.V. Lomonosov Moscow State University, Moscow, 119991, Russia; b Kotel'nikov Institute of Radioengineering and Electronics, (IRE RAS), Moscow, 125009, Russia; c National University of Science and Technology “MISiS”, Moscow, 119049, Russia; d Department of General Physics and Nuclear Fusion, National Research University "Moscow Power Engineering Institute", Moscow, 111250, Russia; e Erich Schmid Institute of Materials Science, Austrian Academy of Sciences, Leoben, 8700, Austria; f NRU South Ural State University, Lenina ave. 76, Chelyabinsk, 454080, Russia; g WPI Advanced Institute for Materials Research, Tohoku University, Sendai, 980-8577, Japan; h Mathematics for Advanced Materials-OIL, AIST-Tohoku University, Sendai, 980-8577, Japan

**Keywords:** Materials science

## Abstract

Fe-based metallic glasses (also called amorphous alloys) are known to have high hardness and high wear resistance. Here we study and present a Fe-Nb amorphous material with an unusual type of electrical conductivity behavior. The electrical transport properties of Fe-Nb oxide layers were studied by measuring local current-voltage characteristics by the atomic-force microscopy technique. At certain voltage levels the samples containing native oxides showed clearly asymmetrical conductivity relative to polarity of the applied potential. Fe-Nb metallic glassy surface oxide film growth process was monitored at ambient conditions. The growth rate keeps constant during the initial 2.5 hours. After that the growth rate drastically decreases and becomes almost zero while the final oxide thickness is 1.0–1.5 nm. The Fe-Nb film sample annealed for 15 minutes at 300 °C demonstrates several times larger oxide thickness and becomes an insulator. X-ray photoelectron spectroscopy was used to characterize the oxidation states in the surface amorphous oxides. This material can be readily applied as inexpensive nanoscale tunnel diode operating at the commonly utilized voltage of ±5 V.

## 1 Introduction

The thin films, representing practical interest, were first produced in middle of the past century. The results and achievements were quickly used in microelectronics and to a smaller degree in engineering. However, the interest in physics and technology of thin films constantly increases. Also, significant progress in composite [1] and layered materials in our days is connected with the studies of thin films and their interfaces.

Metallic glasses [2, 3] and bulk metallic glasses [4, 5] compose an important group of metallic materials which exhibit remarkable properties significantly different from conventional crystalline materials [6]. Metallic glasses can be combined with other materials forming metallic glass/oxide [7] and metallic glass/polymer composites [8]. Recently created nanostructured metallic glasses are also very promising materials for catalytic, chemical and biological applications [9]. A hard refractory metal Nb [10] is one of efficient glass-forming elements improving the glass-forming ability of Ni and Fe [3]. Also, the surface structure of the Ni_62_Nb_38_ (atomic percents) metallic glass was studied and found to consist of the atomic clusters [11, 12].

Successful application of metallic glasses in micro-electro-mechanical devices [13, 14] emphasizes the importance of their surface structure and properties. Characteristic sizes of nano-electro-mechanical systems reach nanometer level [15]. Thickness of a natural oxide on a metallic material is usually a few nanometers [16, 17], which becomes significant when a nano-mechanical element size of several tens or even hundreds of nanometer is used. At the size of the mechanical components of the micrometer and sub-micrometer level, the native surface oxide layer plays an important role in the contact mechanical behavior. The nanoscale tribological behavior and the nanoscale scratch wear resistance of the Ni_62_Nb_38_ (the atomic/molar percents here and elsewhere in the paper) [18, 19], Ti-, Zr- and Mg- [20] and Pt-Cu-Ni-P metallic glasses was studied [21]. The electrical conductivity of natural and artificial oxides of Ni-Nb films [22] was studied and found to be dependent on the oxide type and the layer thickness. Also, electrical properties of the surface oxides, especially a high value of dielectric constant, makes them very promising for creating high-k dielectric capacitors for DRAM memory cells and advanced metal-oxide-semiconductor devices. Fe is located close to Ni in the periodic table and it is somewhat more reactive with oxygen. Replacement of Ni for Fe shall thus cause changes in the composition and properties of the surface oxide film. As well as Zr [23] a refractory metal Nb also increases the glass-forming ability of Fe-based bulk metallic glasses [24]. Contrary to the Ni-Nb system in which the lowest temperature eutectic is at 40.5 at.% Nb the Fe-Nb system has the lowest temperature eutectic at 12.1 at.% Nb.

Fe-based metallic glasses (also called amorphous alloys) are known to have high hardness of about 700–1000 HV [25, 26] and high wear resistance [27]. In the present work we study the surface structure and properties of the Fe_86_Nb_14_ metallic glass film produced under conditions determined in [28] for the formation of smooth metallic films.

## 2 Experimental

The films were prepared by magnetron sputtering from a Fe target with Nb pieces on top of it. The addition of Nb induced formation of the amorphous material. The substrate, a pure copper sheet, was polished prior to deposition. The final Fe-Nb film thickness was about 100 nm. A separately prepared film also annealed directly in the deposition chamber at 300 °C for 15 min in a dry mixture of nitrogen (75 %) and oxygen (25 %) gases.

X-ray diffractometry with a characteristic Cu Kα radiation was used to study the phase composition. The chemical composition was measured by energy-dispersive X-ray analysis in a scanning electron microscope (SEM). The measured film composition was Fe_86_Nb_14_.

The X-ray photoemission spectroscopy (XPS) studies of the samples surfaces were performed with the help of the electron-ion spectroscopy module based on Nanofab 25 (NT-MDT) platform. In the analysis chamber an ultrahigh oil-free vacuum about 10^−7^ Pa. The X-ray source SPECS XR 50 without a monochromator with Mg anode as the X-ray source (1253.6 eV photon energy) was used. The spectra were recorded with an electrostatic hemispherical energy analyzer SPECS Phoibos 225. The energy resolution based on the full width at half maximum (FWHM) of the spectrometer at the Ag3d_5/2_ line (peak) was 0.78 eV for non-monochromatic X-radiation Mg Kα. The energy positions of the spectra were calibrated with reference to the Cu2p_3/2_ (binding energy 932.62 eV), Ag3d_5/2_ (368.21 eV) and Au4f_7/2_ (83.95 eV) peaks. All survey spectra scans were recorded at a pass energy of 80 eV. The detailed scans of strong lines were in most cases recorded as wide as needed just to encompass the peak(s) of interest and were obtained with a pass energy of 20 eV. Non-destructive chemical and phase depth profiling of nano-sized films in this investigation was carried out on the base of method [29]. This method enables to determine the depth profiles with a sub-monolayer accuracy using XPS data.

Surface topography and local current-voltage (I-V) characteristics (CVC) were measured using atomic force microscope AIST-NT SPM (model SmartSPM-1000) at ambient conditions. Tapping mode of the atomic force microscopy (AFM) technique was used for taking topography profiles and contact mode was used for taking a series of local CVC's. Pt coated conductive cantilevers (Microscience, model N14/Pt) with a spring constant ranged from 6 to 10 N/m and the cantilever tip radius <40 nm were used for CVC measurements. The diamond single crystal cantilevers (AFM Probe D300, SCDprobes) with spring constant of 40 N/m and 5–10 nm radius were used for the tribological experiment. The tribological properties of the oxide film were studied by nano-scratch test with the experimental setup used in Ref. [18]. The spring constant was measured for each cantilever using a technique based on measuring the change in resonant frequency of the fundamental mode of vibration [30].

CVCs were recorded with the voltage sweeping rate of 1 ms/point, while digital feedback kept constant AFM cantilever load of 20 nN during all measurements. The topography profiles were recorded for long time to keep exactly the same region of CVC recording. Bulk resistivity of the film measured by the van der Pauw technique [31] was purely metallic and the I-V curves were linear. For removing oxide layer, the surfaces of the samples were finely polished by the 0.25 μm particle size diamond paste.

## 3 Results and discussion

**Fig. 1:**
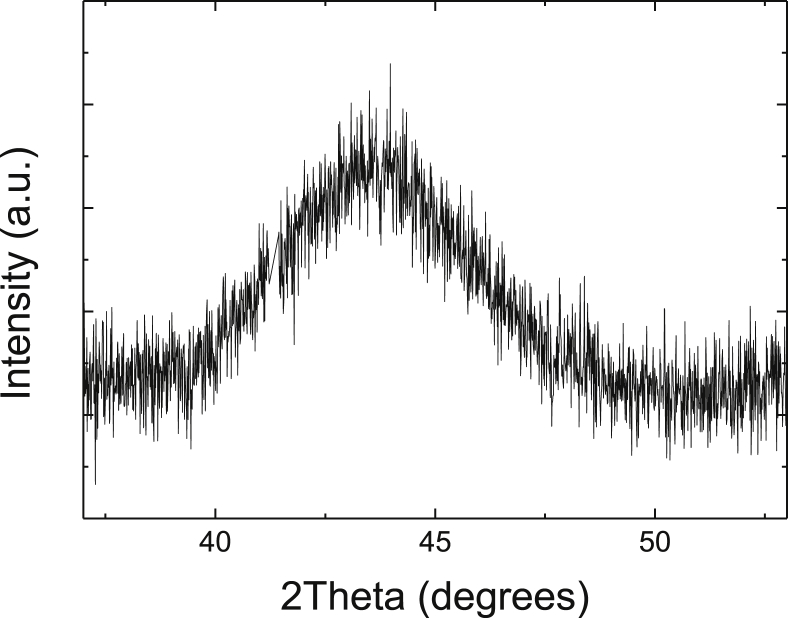
X-ray diffraction pattern of the Fe_86_Nb_14_ film.

X-ray diffraction showed that the film had a fully amorphous structure (Fig. 1) without any sharp peaks of a crystalline phase(s). The first broad diffraction maximum is located at about 43 degrees of 2θ which is typical for Fe-based metallic glasses [32]. It is understood as Nb is one of effective elements improving the glass-forming ability of Fe [23].

**Fig. 2:**
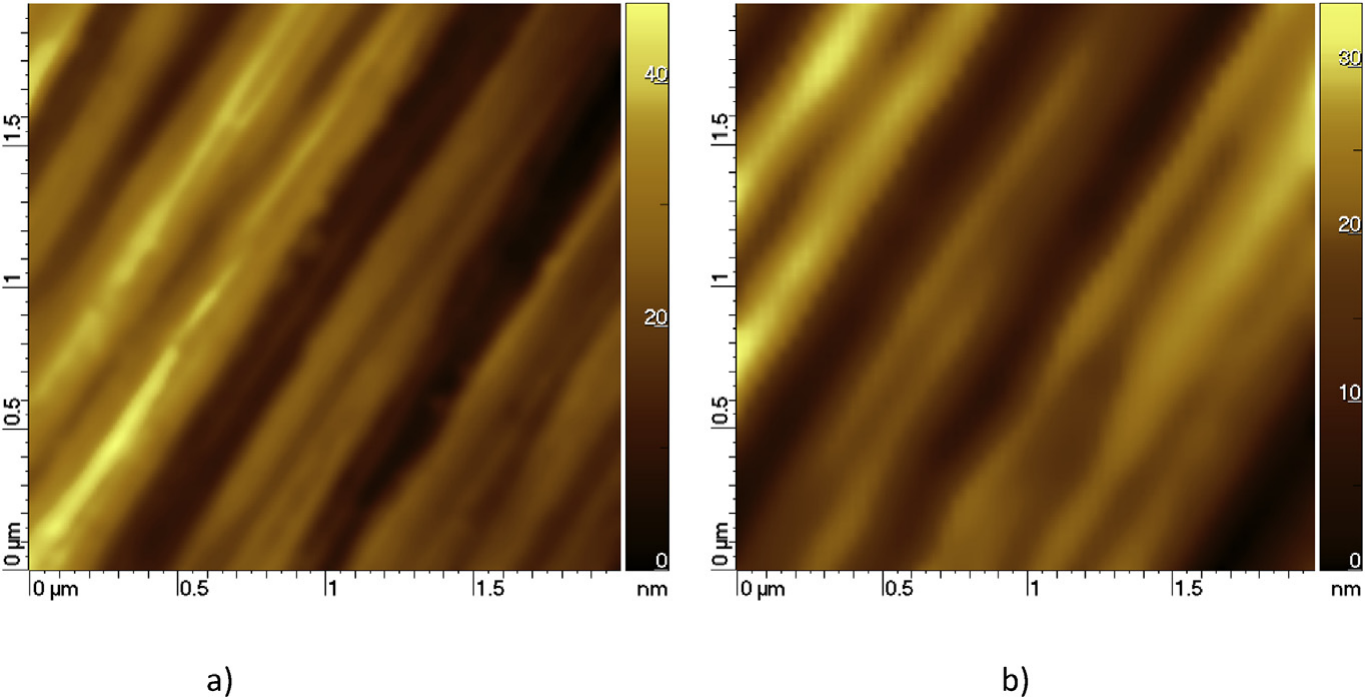
AFM topography of the Fe-Nb film with the native oxide (a) and annealed at 300 °C (b).

AFM topographies of the natural and artificial oxide surface are shown in Fig. 2. Typical average roughness (R_a_) of the surface was 12 ± 2 nm for the sample with native oxide and 10 ± 2 nm for the annealed samples of 4 × 4 μm area. Relatively high roughness values are due to the scratches on the base copper substrate necessary to measure electrical conductivity.

The Fe-Nb sample has a greater wear resistance compared with the Ni-Nb one [17]. Under a load of 5 μN, the single pass scratch depth of the Fe-Nb sample was less than 0.3 nm corresponding to the wear coefficient (k_w_) was less than 0.5 nm^2^/μN.

**Fig. 3:**
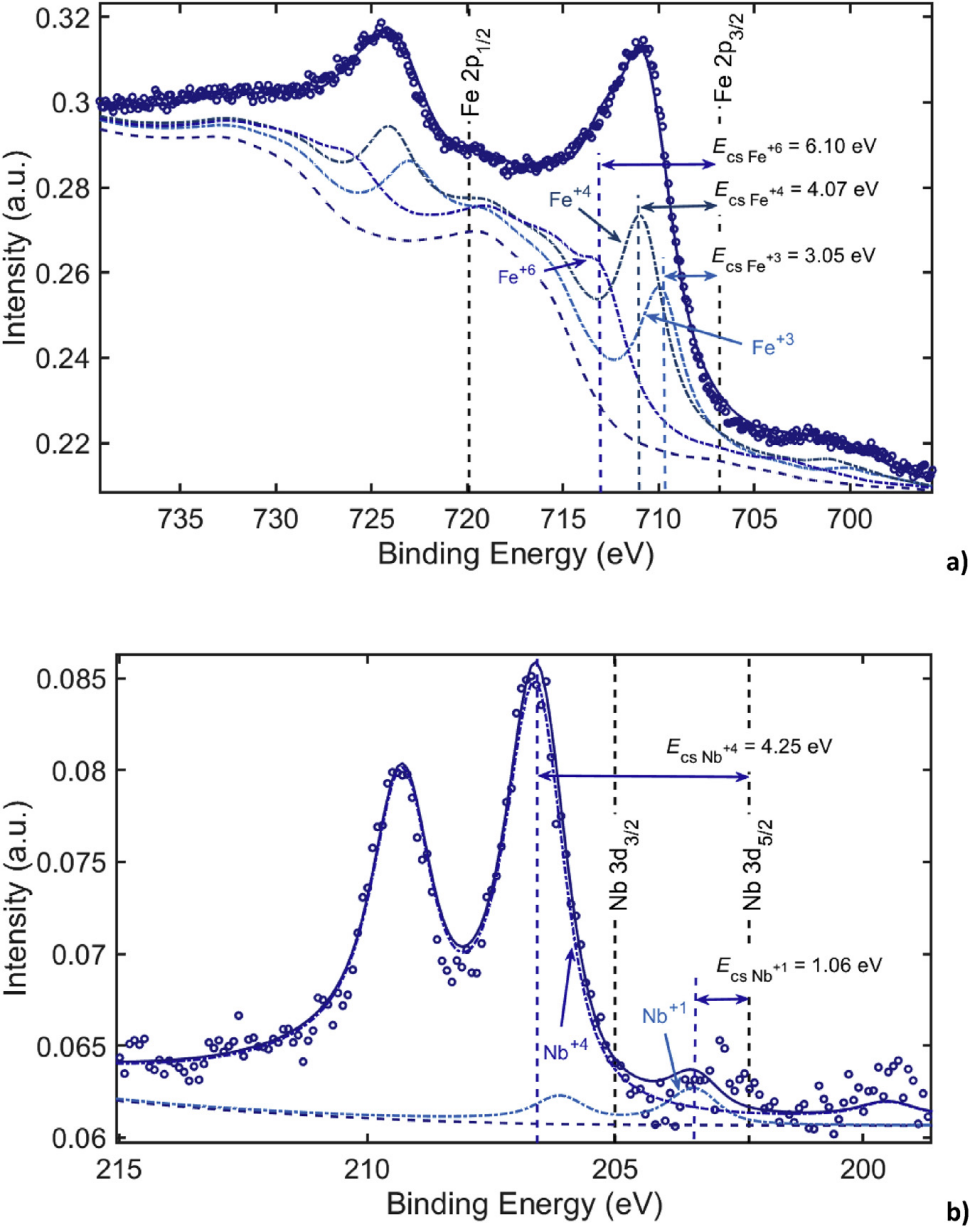
XPS spectra: a) Fe2p and b) Nb3d.

The obtained XPS spectra are shown in Fig. 3. Circles show recorded detailed spectra, the solid lines: calculated using method [29], the dotted line: separate calculated peaks. Information on chemical and phase composition on the layers was obtained by the analysis of peaks produced by elastically scattered electrons. Surface of the samples was found to be natively oxidized in air. The chemical shift energy $E_{\text{cs}}=zE_{\text{cs}1}$ depends almost linearly on the oxidation rate z of the element, here $E_{\text{cs}1}$ is the chemical shift energy per unit of oxidation rate. To find $E_{\text{cs}1}$ of lines Nb3d and Fe2p, we used the values of chemical shifts for oxides ${\text{Nb}}_{2}{\text{O}}_{5}$ and ${\text{Fe}}_{2}{\text{O}}_{3}$: $E_{{\text{cs}\;\text{Nb}}_{2}{\text{O}}_{5}}=5.31$ eV and $E_{{\text{cs}\;\text{Fe}}_{2}{\text{O}}_{3}}=3.05$ eV [33]. Fig. 3 presents the chemical shift energies for partial peaks. The line decomposition procedure [29] showed that line Fe2p was decomposed into three separate peaks corresponding to oxidation rates of Fe: +3, +4, +6; and line Nb3d into two peaks: +1, +4. The +6 oxidation rate of iron possibly corresponded to ferrate anion ${\text{FeO}}_{4}^{-2}$. As far the films were thin it was hard to define any phase of oxide: probably these were complex oxides Fe-Nb. As defined from the XPS spectra the oxide layer composition was: 0.46(Fe^+4^) + 0.31(Fe^+3^) + 0.21(Nb^+4^) + 0.01(Nb^+1^) (upper indexes indicate the oxidation state). Its thickness was determined to be 1.2 ± 0.2 nm. The relative atomic concentrations of the constituent metallic elements determined using the XPS spectra were 90 ± 2 at.% for Fe and 10 ± 2 at.% for Nb. The XPS measurements indicate that the surface composition is close to that of the metallic glassy phase and both Fe and Nb elements are oxidized. Fe indicates its typical oxidation number of +3 and less common number of +4 while the oxidation number of Nb is lower than +5 which is a typical value of for this element.

**Fig. 4:**
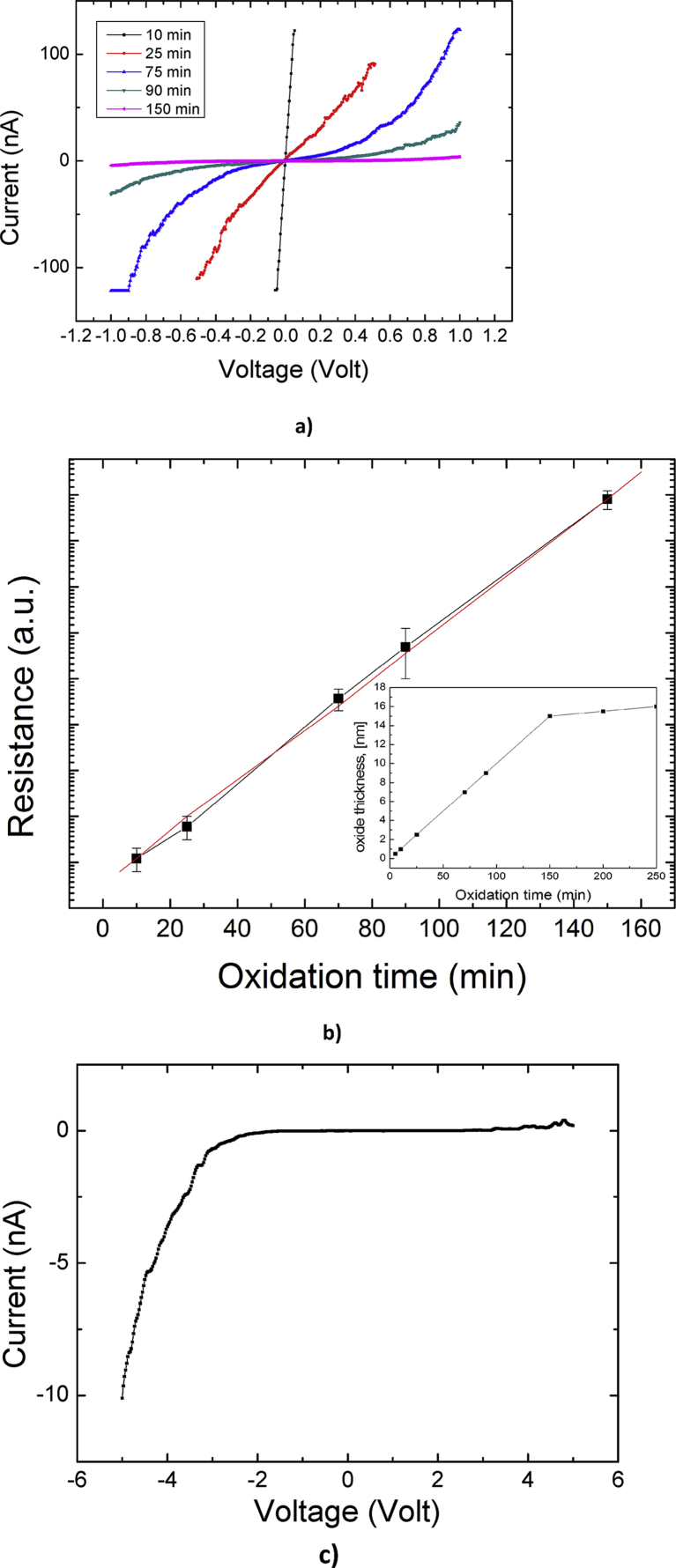
a) time evolution of CVCs after polishing; b) evolution of the electrical resistance values with time after polishing (black dots). Red curve shows linear fit; Inset shows calculated oxide thickness on time; c) CVC recorded in the wide range of voltages from -5 to +5 V after 180 minutes of surface oxidation at ambient condition indicating asymmetrical conductivity.

Electrical transport properties of Fe-Nb oxide layers were studied by measuring local CVCs by the atomic-force microscopy technique. The family of CVC traces measured in the voltage range from -1 to +1 V immediately after the naturally oxidized surface was polished is shown in Fig. 4a. Initially a linear metallic conductivity is observed. Further, as the oxide layer grows, the CVC shape becomes nonlinear. The shape of the CVC curves is symmetrical at positive and negative voltage. The estimated from CVC differential resistance growth rates of the natural oxide film on the Fe-Nb surface at ambient conditions are 0.4–0.6 nm/hour. The growth rate keeps constant for the initial 2.5 hours. After that the growth rate drastically decreases and becomes almost zero leading to the final oxide thickness of 1.0–1.5 nm which is in good agreement with the XPS data. The Fe-Nb film sample annealed for 15 minutes at 300 °C demonstrates several times larger oxide thickness.

The evolution of the resistance of the oxide on time is shown in Fig. 4b. It can be seen that for the first 150 minutes after polishing, the electrical resistance of the platinum tip electrode/oxide/Fe-Nb contact changes linearly in logarithmic scale. Later, the resistance of the film remains practically unchanged. The electrical current in structure of the platinum/oxide/Fe-Nb can be described with good accuracy by the Simmons model for electric tunnel effect through a potential barrier of arbitrary shape existing in a thin insulating film [34]. For low voltages current density *J* is exponentially depends on mean barrier height $\bar{\varphi }$ (Eqs. (1) and (2)):

(1) $$J=J_{L\;}{\bar{\varphi }}^{1/2}\;V\;exp\left(-A\varphi^{-1/2}\right)$$

where

(2) $${J_{L\;}=\left[\frac{{\left(2m\right)}^{\frac{1}{2}}}{\text{Δ}s}\right]\left(\frac{e}{h}\right)}^{2}$$

Here *V* - voltage across the structure, *A* – a constant, *m* - the mass of electron, *e* - the charge of electron, *h* - the Planck's constant and *Δs* - the difference of limits of barriers at the Fermi level.

When measuring CVC over a wider range of voltages from -5 to +5 V after 180 minutes of oxidation, there is a sharp increase in current at negative voltages with a threshold around -3 V (Fig. 4c). The increase in current is due to the suppression of the tunnel barrier by an electric field. The absence of conductivity at large positive voltages indicates the absence or impossibility of formation of n-type charge carriers in the Fe-Nb oxide film. Apparently, the structure of the natural Fe-Nb oxide film consists of an upper layer of Fe-NbO_x_ and a ferrum-enriched lower layer, which provides n-type conductivity. The system can be regarded as consisting of two Schottky barriers (Pt/(Fe,Nb)O_x_/FeNb metallic matrix), separated by the FeNb oxide tunnel barrier. According to the observed properties these materials can be applied as inexpensive nanoscale tunnel diodes operating at the commonly utilized voltage of 5 V. It is also important to note that the electrical properties of a material can be drastically varied in a wide range by native or artificial oxidation. Selective oxidation and patterning on a metallic glassy surface was recently made by laser irradiation [35]. This process can be applied for current alloy to produce local surface areas with the required electrical properties of the present Fe-Nb samples. Surface oxidation and amorphization was obtained by dealloying which is another suitable method of production of surface materials [36] and can also be applied to current material to modify its surface properties. The polished Fe-Nb film annealed for 15 minutes at 300 °C becomes an insulator (contact resistance is greater than 2 TOhm), which corresponds to an oxide film thickness greater than 5 nm.

## 4 Conclusion

The electrical transport properties of Fe-Nb oxide layers were studied by measuring local current-voltage characteristics by the atomic-force microscopy technique. The samples containing native oxides showed a clearly asymmetrical conductivity. It was found that natural oxide film grows at about 0.4–0.6 nm/hour on the Fe-Nb metallic glassy surface at ambient conditions. The growth rate keeps constant for initial 2.5 hours. After that the growth rate drastically decreases and becomes almost zero and final oxide thickness is 1.0–1.5 nm. The Fe-Nb film sample annealed for 15 minutes at 300 °C demonstrates several times larger oxide thickness and becomes an insulator. X-ray photoelectron spectroscopy showed that surface oxide corresponds to the oxidation states of Fe^+4^, Fe^+3^ and Nb^+4^ which may indicate a mixture of Fe and Nb amorphous oxides. These materials can be applied as inexpensive nanoscale tunnel diodes operating at the commonly utilized voltage of 5 V.

## Declarations

### Author contribution statement

A. S. Trifonov, A. V. Lubenchenko, D. V. Louzguine-Luzgin: Conceived and designed the experiments; Performed the experiments; Analyzed and interpreted the data; Wrote the paper.

S. V. Ketov: Conceived and designed the experiments; Performed the experiments; Analyzed and interpreted the data.

S. V. Taskaev: Conceived and designed the experiments; Analyzed and interpreted the data.

### Funding statement

This work was supported by the World Premier International Research Center Initiative (WPI), MEXT, Japan and by Act 211 Government of the Russian Federation (contract no. 02.A03.21.0011).

### Competing interest statement

The authors declare no conflict of interest.

### Additional information

No additional information is available for this paper.
